# Lung Protection vs. Infection Resolution: Interleukin 10 Suspected of Double-Dealing in COVID-19

**DOI:** 10.3389/fimmu.2021.602130

**Published:** 2021-03-03

**Authors:** Holger A. Lindner, Sonia Y. Velásquez, Manfred Thiel, Thomas Kirschning

**Affiliations:** Department of Anesthesiology and Surgical Intensive Care Medicine, Medical Faculty Mannheim, University Medical Center Mannheim, Mannheim Institute for Innate Immunoscience (MI3), Heidelberg University, Mannheim, Germany

**Keywords:** viral clearance, SARS-CoV-2, interleukin 10, COVID-19, endotype, lung

## Abstract

The pathological processes by severe acute respiratory syndrome coronavirus 2 (SARS-CoV-2) infection that make the virus a major threat to global health are insufficiently understood. Inefficient viral clearance at any stage is a hallmark of coronavirus disease 2019 (COVID-19). Disease severity is associated with increases in peripheral blood cytokines among which interleukin 10 (IL-10) increases particularly early and independent of patient age, which is not seen in active SARS-CoV infection. Here, we consider the known multi-faceted immune regulatory role of IL-10, both in protecting the lung from injury and in defense against infections, as well as its potential cellular source. While the absence of an IL-10 response in SARS is thought to contribute to early deterioration, we suspect IL-10 to protect the lung from early immune-mediated damage and to interfere with viral clearance in COVID-19. This may further both viral spread and poor outcome in many high-risk patients. Identifying the features of the viral genotype, which specifically underlie the different IL-10 dynamics as an etiological endotype and the different viral load kinetics and outcomes as clinical phenotype, may unveil a new immune evasive strategy of SARS-CoV-2.

## Introduction

Research into coronavirus disease 2019 (COVID-19) has reached an unprecedented scale since the beginning of the severe acute respiratory syndrome coronavirus 2 (SARS-CoV-2) pandemic. It provides the foundation for public health protection measures and clinical management of infected patients. Yet, the particular mechanisms that account for a requirement for hospitalization in as much as 20% of infected individuals (1), and over 2.3 million deaths globally attributed to COVID-19 at the time of writing (2) remain insufficiently understood (3). In the following, we first consider the characteristics of the immune response to SARS-CoV-2 and of its transmission. Then, we highlight patient studies on COVID-19 that identified increased blood levels of the major anti-inflammatory cytokine interleukin 10 (IL-10) that display a dynamic pattern different from other cytokines and not seen in SARS. We summarize the evidence for the potential of IL-10 to protect lung tissue from immune-mediated damage but also to impede antimicrobial defense, and consider possible cellular sources of IL-10. To illustrate the etiological role of IL-10 in COVID-19 we finally propose a viral genotype-endotype-clinical phenotype relationship.

## Characteristics of the Immune Response to SARS-CoV-2

One of the hallmarks of COVID-19 pneumonia is an association of viral load and disease severity with an inflammatory cytokine response detectable in peripheral blood (4–11). Although moderate compared to other causes of acute critical illness including acute respiratory distress syndrome (12, 13), this response has been invoked to explain many features of SARS-CoV-2 pathophysiology, to predict patient outcome and to guide therapeutic strategies (14–17). For example, elevated tumor necrosis factor alpha (TNF-α) and interleukin 6 (IL-6) are presumed to contribute to lymphocytopenia and elevated IL-10 to impaired antigen-presentation and co-stimulation capacity of macrophages and dendritic cells as well as exhaustion of T cells (18–20).

Similar to SARS-CoV (21), critical SARS-CoV-2 infection dampens the antiviral type 1 interferon (IFN) response detectable in peripheral blood (10) despite the presence of an inflammatory blood monocyte population with yet a strong IFN stimulated gene (ISG) signature in mild COVID-19 (22). In the respiratory tract of COVID-19 patients, increased type 1 IFN gene expression was still detected in upper and lower airway fluids (10, 23) as well as a robust ISG response in differentiating airway epithelial cells (23), in bronchoalveolar lavage (BAL) (24) and, specifically, in BAL neutrophils (25). In an autopsy study on a cohort of 16 COVID-19 patients that died from respiratory failure, targeted gene expression profiling in lung tissue samples assigned seven patients each to a group with high and with low ISG expression (26). Survival time from hospitalization was significantly shorter in the ISG^high^ than in the ISG^low^ group (approximate median of 4 vs. 9 days). Besides an earlier death, high ISG expression was also associated with high levels of pro-inflammatory cytokines, high copy numbers of viral RNA, and relatively intact lung morphology. By contrast, a later death and low ISG expression were associated with lymphocyte and macrophage infiltration, complement activation, and diffuse alveolar damage. Nienhold et al. suggest that these two distinct immunopathological profiles represent sequential phases of COVID-19, each with a high mortality risk (26).

Changes to immune cell subsets in peripheral blood of COVID-19 patients also resemble SARS-CoV infection (27). Lymphocytopenia is associated with poor outcome (28). Numbers of circulating dentritic cells (10, 29, 30) and eosinophils (31) were likewise reduced. Functionally, helper and cytolytic T cells as well as natural killer (NK) cells appeared exhausted (19, 32), and the relative fraction of naïve helper T cells was increased at the expense of memory helper T cells (11). In cultured peripheral blood mononuclear cells (PBMCs) from critically ill COVID-19 patients, anti-CD3/anti-CD28 antibody stimulated IFN-γ production in lymphocytes and endotoxin stimulated TNF-α production in myeloid cells were reduced at least by half. This was observed not only in comparison to healthy controls but also to critically ill patients with sepsis by other infections and without sepsis (30).

In the respiratory tract, single-cell transcriptomics identified enrichment of cytotoxic lymphocyte subsets and NK cells in the upper airways of patients with moderate and severe COVID-19 (33) and of NK cells in BAL from severe cases (25). In the same BAL dataset, an independent pathway analysis in genes differentially expressed in CD8^+^ T cells from COVID-19 patients suggests, however, impaired clonal expansion and effector phenotype in severe compared to moderate disease but high clonal expansion in moderate disease (34). This is in line with elevated frequencies of programmed cell death protein 1 positive CD8^+^ T cells in the late death ISG^low^ lungs in the autopsy study by Nienhold et al. (26).

Last but not least, analyses of thoracic lymph nodes and spleen in fatal COVID-19 demonstrated the loss of germinal center (GC) B cells, follicular T helper cells and GCs themselves (35), which again is reminescent of SARS (36). Nevertheless, activation of extrafollicular, i.e., blood, B cells correlated with the expansion of class-switched antibody-secreting cells and high titers of SARS-CoV-2-neutralizing antibodies in critical illness with poor outcomes compared to mild COVID-19 (35).

Taken together, compartmentation and interplay of innate and adaptive immunity in COVID-19 appear dysregulated, not unlike what was described for SARS (37). This dysregulation is already apparent from peripheral blood immune cell, cytokine and metabolite profiles in patients with moderate compared to mild disease, and it seems further increased in severe disease (38). Failure of innate and adaptive immunity to cooperate in the control of the virus on the one hand and in the resolution of the inflammation on the other may drive early and late mortality, respectively, in accordance with the two-phase model of Nienhold et al. (26). The importance of detrimental hyperinflammation in the later phase is underscored by the overall reduction in 28-day mortality from 41.5 to 32.7% in a meta-analysis of severely ill COVID-19 patients randomized to temporary corticosteroids (39).

An important question is to what extent natural and vaccinal immunity provide lasting protection against COVID-19 and prevent further SARS-CoV-2 transmission. With efficacies at over 94% and no serious safety concerns in phase 3 studies, the two RNA vaccines BNT162b1 (40) and mRNA-1273 (41), encoding the receptor-binding domain (RBD) of the SARS-CoV-2 spike (S) protein, have spearheaded the development of SARS-CoV-2 vaccines (42) and the first vaccination campaigns. Yet, the contributions of natural and vaccinal immunity to SARS-CoV-2 to population immunity will only emerge over time (43). The following considerations on SARS-CoV-2 transmission vs. clearance are consistent with an important role of viral immune evasion in curbing immunity and favoring the pandemic levels of spread.

## SARS-CoV-2 Transmission vs. Clearance

SARS-CoV-2 transmission is well-documented not only for symptomatic but also for asymptomatic, pre-symptomatic and convalescent patients with mild or moderate disease (44–48). The extent of transmission through pre-symptomatic and symptomatic infections is thought to be comparable (49, 50). Yet, transmission modeling suggests that asymptomatic individuals have represented the major source of infections so far (51, 52). However, this has to be viewed in the light of the methodological challenges in the estimation of asymptomatic spread (53). Transmission in the absence of symptoms, whether asymptomatic or pre-symptomatic, has been coined silent transmission (54).

To explain the high frequencies of pre-symptomatic transmission of SARS-CoV-2, and potentially silent transmission overall, it is instructive to consider emerging differences in viral load dynamics and duration of infectivity for this virus compared to other respiratory viruses including SARS-CoV. The median incubation time of SARS-CoV-2 is estimated at around 5 days (55) compared to 4 days for SARS-CoV, only 3.2 days for endemic human coronaviruses and 2.6, 1.9, 1.4, and 0.6 days, respectively, for parainfluenza virus, rhinovirus, influenza virus A (IVA) and IVB (56). A preprint on the modeling of viral load trajectories (57) supports the notion that viral load peaks before symptom onset for SARS-CoV-2 (49) but only thereafter for SARS-CoV (58) with longer subsequent median shedding durations for SARS-CoV-2 (4.8 days) than for SARS-CoV (1.2 days). Together, these observations are overall consistent with a longer duration of infectivity from pre-symptomatic to convalescent spread in SARS-CoV-2 infection compared to SARS-CoV and other respiratory viruses (46).

Patients with severe and critical COVID-19 may even shed virus beyond day 9 or 10 of symptom onset (59, 60). A persistently high SARS-CoV-2 titer is a marker for disease severity and prognosis (28), and post-mortem RT-PCR detection of viral RNA in the respiratory tract can ascertain death from COVID-19 (61). Together, the transmission characteristics and the high persistence of SARS-CoV-2 indicate limited efficiency in viral clearance at any stage of the infection as well as any degree of disease severity. Next, we introduce reported observations that lead us to propose that the anti-inflammatory cytokine IL-10 plays a role in an underlying immune evasion strategy of SARS-CoV-2.

## IL-10 in COVID-19

Compared to adults, children are less affected by COVID-19 (62, 63), although SARS-CoV-2 titers in their upper airways are similar (64, 65), and infants still appear particularly vulnerable to SARS-CoV-2 infection (66) and critical disease (67). In addition to clinical characteristics, two studies also assessed immune features in infected children. Sun et al. retrospectively examined laboratory test results for six cytokines in the blood of 26 infants aged <1 year (excluding newborns) and treated for COVID-19 in the Wuhan Children's Hospital (68). They found increased admission levels of IL-4, IL-6, TNF-α and interferon γ (IFN-γ) in <20%, but of IL-10 in 50% of the patients. IL-2 levels were normal in all patients. In 157 pediatric patients with mild and moderate COVID-19 disease from the same hospital, admission levels of the same cytokines did not differ, except for the median concentration of IL-10 (3.58 vs. 3.96 pg/mL with a concurrent median age difference of 108 vs. 66 months) (69). The data from both studies very likely originated from the same clinical laboratory and from potentially slightly overlapping patient populations (68, 69). Nevertheless, they suggest that IL-10 is the only of these cytokines for which an increase is associated with pediatric COVID-19 whereas increases of all six are seen in adult patients (4–11).

In a longitudinal study in 71 adults, IL-10 and IL-1 receptor antagonist (IL-1RA) were the only among a panel of 34 immune mediators that were increased as early as in the first week following the onset of clinical COVID-19 symptoms in those who developed severe disease (18 patients) beyond the second week compared to continued moderate disease (53 patients) (9). In experimental rhesus macaques, serum IL-10 levels were elevated about 16-fold even as early as on the first day after SARS-CoV-2 infection. This increase was almost fully repressed by the adenovirus-vector-based vaccine AZD1222 compared to control-vaccinated animals (70).

Several studies in Chinese cohorts from the very beginning of the pandemic found that blood levels of IL-10 correlated with severity of COVID-19 along with most other cytokines (4, 6, 11), while a subsequent study from France found no significant differences between mild/moderate, severe, and critical cases (10). In a recent characterization of the cytokine response in COVID-19 patients hospitalized in Ireland, who were stable and who required ICU admission 1 week after the onset of symptoms (20 patients per group), IL-10 was also equally elevated in both groups, while IL-1β, IL-6, IL-8, and soluble TNF receptor 1 were all more strongly elevated in the ICU than in the stable group (71).

Following symptom onset (7, 72) or hospital admission (73), blood levels of IL-10 were reported to slowly decline over the course of 2–3 weeks or when symptoms declined (18). However, data on COVID-19-convalescent blood has not yet been reported explicitly.

In contrast to COVID-19, blood levels of IL-10 in symptomatic SARS patients, including severe disease, did not differ from control blood (74–76) but were markedly increased only in convalescent patients (77). Such a late increase agrees with expectations because IL-10 is well known to support resolution of inflammation and tissue repair and to protect from tissue damage in autoimmune diseases (78, 79) including lung damage as set out below. Notably, the lack of an increase in IL-10 in SARS-CoV infection has been suggested to contribute to immune-mediated lung damage early on (75) and, more recently, to the higher frequency of fatal aggravation of lung injury in SARS compared to COVID-19 (80).

Taken together, COVID-19 is characterized by a more consistent increase in blood IL-10 across the age groups of patients and an earlier onset of its increase than seen for other blood cytokines, as well as a possibly similar increase in stable and critical illness and a slow decline following symptom onset. This particular IL-10 dynamics in COVID-19 markedly differs from SARS where plasma IL-10 remained unchanged in active disease and increased only in convalescent patients.

## Ambiguous Role of IL-10 in Lung Injury and Infection

Lung protective activity has been experimentally demonstrated for IL-10 in animal models of endotoxemia (81), mechanical ventilation (82), hyperoxia (83), induced asthma (84), transplantation (85), and IVA and *Pseudomonas* infections (86, 87). In mouse models of IVA pneumonia, IL-10 produced by effector T cells protected from immune-mediated lung damage (87) but also interfered with protection through a virus specific T helper 17 cell response against a lethal dose IVA challenge (88) and inhibited antimicrobial immunity upon subsequent pneumococcal infection (89–92). In humans, IL-10 has likewise been linked to tissue protection against an exacerbated antimicrobial immune response in bacterial, viral and also parasitic infections but at the same time to microbial persistence inside as well as outside of the lung (93–96).

Notably, pulmonary IL-10 is thought to abet persistence of the world's most prevalent bacterial infection, *Mycobacterium tuberculosis* (*Mtb*) (97). Motivated by a reported epidemiological relationship between active tuberculosis and fatal pandemic influenza infections in South Africa, a recent study investigated the effects of IVA challenge and IL-10 signaling on bacterial load in *Mtb* infected mice (98). A significantly increased *Mtb* burden in the lungs through IVA coinfection could indeed be reduced to *Mtb*-only control levels by antibody-mediated IL-10 receptor blockade, yet, in a T cell independent manner. A possible implication is that promotion of *Mtb* persistence by IL-10 concomitantly increases the risk of death from influenza.

The ambiguous role of IL-10 is further exemplified by its ability to induce IFN-γ in a murine model of central nervous system (CNS) infection with the neurotropic strain of mouse hepatitis virus (MHV), a coronavirus (99). IL-10 has been shown to be able to both augment cellular cytotoxicity against MHV infected cells through IFN-γ, improving outcome, and to protect from neuronal damage by dampening the adaptive immune response, concurrently, promoting chronic infection including neurotrophic coronavirus encephalomyelitis (99). Notably, SARS-CoV-2 is also neurotropic (100), but severity of neuropathology does not appear to correlate with presence of the virus in the CNS and may rather be mediated by neuroinflammation in the brainstem (101).

As another example for the ambiguity in IL-10 function, a recent study by Mazer et al. illustrates that IFN-γ may mediate both immune stimulatory and inhibitory effects of IL-10 in human sepsis (102). PBMCs derived from critically ill sepsis patients but not from critically ill and from healthy controls released IFN-γ in response to treatment with either IL-10 or an IL-10 inactivating antibody. To reconcile their seemingly contradictory observations, they proposed that sepsis CD8^+^ T cells became poised to respond to IL-10 with IFN-γ production on the one hand, and that inactivation of regulatory T cell (Treg) derived IL-10 unleashed IFN-γ release from Th1 cells on the other (102). In the following, we consider cellular sources of IL-10 and the evidence for IL-10 producing cells in COVID-19 patients.

## Cellular Sources of IL-10

Protection from tissue damage during autoimmune reactions through IL-10 producing Treg cells and autoregulatory T helper type 1 (Th1) cells, including in the lung, is a long-standing concept (103, 104). The potential of IL-10 producing, virus-specific Tregs, in particular, in the treatment of human coronavirus-induced demyelinating disease has been discussed recently (105, 106). In addition to Tregs, other immune cell populations can contribute to IL-10 production constitutively or in a dynamic fashion during infections. IAV infection, for instance, primed different immune cell populations in the mouse lung to produce IL-10 in response to *ex vivo* phorbol ester/ionomycin treatment (98). Increased proportions of IL-10 positive innate immune cells (dendritic cells, neutrophils and NK cells) were detected 3 days post-inoculation while cytotoxic and helper T cells dominated by day nine. Overall, it emerges that in the acute phase of viral infections, IL-10 release from dendritic cells, neutrophils, NK cells and effector T cells balances immune damage and defense (107).

In human peripheral blood, eosinophils are constitutive producers of IL-10 (108). In mouse lung, interstitial macrophages (IMs) are an important constitutive source of IL-10 (109) and were protective in mouse models of allergic lung inflammation (84, 110, 111). Resting and endotoxin stimulated human IMs secreted larger amounts of IL-10 and also IL-1RA and IL-6 than alveolar macrophages (AMs) *ex vivo* (112). Recently, a morphologically distinct population of IMs was described in humans and mice that is localized around the large bronchiolar airways and in association with sympathetic fibers and was thus referred to as nerve- and airway-associated macrophages (NAMs) (113). Upon treatment with IAV or polyinosinic:polycytidylic acid [poly(I:C)], mouse NAMs proliferated and became a major source of IL-10, unlike AMs that, by contrast, engulfed virus particles. The authors suggested that NAMs are critical for lung tissue homeostasis in both the steady state and following inflammatory stimuli (113). Last but not least, a regulatory subset of peripheral B cells, characterized by the ability to produce IL-10 upon *ex vivo* treatment with phorbol ester/ionomycin, is increased in adult autoimmune diseases (114).

The cellular source of increased IL-10 in blood of COVID-19 patients as well as IL-10 protein levels in the SARS-CoV-2 infected lung have not been reported so far. Flow cytometric analysis of the T cell compartment in *ex vivo* stimulated PBMCs of COVID-19 patients revealed a subpopulation of IL-10 producing Treg cells that amounted to 2% of the total Treg population in healthy controls, to 6% in mild-moderate and to 10% in severe COVID-19 disease, notably, in the absence of other significant differences (115).

To eventually assess the lung protective potential of IL-10 in COVID-19, it will be crucial to elucidate whether its increase in blood is an indirect sign of its production in the lung or arises in the periphery. Here, we considered lung IMs, including NAMs, as a possible pulmonary source. Analyses of immune cells in BAL from COVID-19 patients by single-cell sequencing suggests that recruited inflammatory monocytes, neutrophils, and macrophages account for the vast majority of myeloid cells in lung alveoli of severe cases (25, 28). NK cells were enriched in BAL from severe cases (25) and NK cells and cytotoxic lymphocytes in the upper airways of moderate and severe cases (33). Eosinophils were reportedly not enriched in lung tissue in two post-mortem examinations (116). As set out above, any of these immune cell populations could, in principle, also produce IL-10.

## Discussion

### IL-10 Suspected of Double-Dealing in COVID-19

We reviewed clinical observations in SARS-CoV-2 and SARS-CoV infected patients and the roles of the anti-inflammatory cytokine IL-10 in experimental animal models of lung injury and infection as well as in human pulmonary pathology and infection. Here, we present reasons to suspect that IL-10 actively contributes to COVID-19 pathology by impeding resolution of SARS-CoV-2 infection rather than representing a bystander. Its early increase in blood of COVID-19 patients from symptom onset to severe and critical illness thus merits the same attention as other altered cytokines.

Despite a lower mortality rate in COVID-19 than SARS, the immune response to SARS-CoV-2 shows many similarities to the one described for SARS-CoV. As a remarkable difference though, blood levels of IL-10 are consistently elevated in active COVID-19 but remained unchanged in SARS, where levels did not rise until convalescence. In particular, IL-10 was found increased more consistently than other cytokines in pediatric COVID-19 at hospital admission and earlier than most other cytokines in symptomatic adult patients, where its levels may show an association with disease severity. We considered the characteristic anti-inflammatory and tissue protective properties of IL-10 in lung and CNS on the one hand and its ability to inhibit antimicrobial immunity in the lung and to further chronification including of coronavirus CNS infection and tuberculosis on the other. IL-10 also displays ambiguous behavior in murine neurotropic coronavirus encephalomyelitis and human sepsis through opposing effects on IFN-γ production. The multi-faceted nature of IL-10 likely also applies in COVID-19.

Here, we put forward the hypothesis that the early increase in IL-10 in COVID-19 contributes to more efficient viral spread compared to SARS. The observation that, in SARS-CoV-2 infections, IL-10 increased as early as in the first week after symptom onset in patients that subsequently developed severe disease (12) raises the question whether IL-10 modifies the course of the infection already before symptom onset when viral loads peak, while SARS-CoV load peaks not until after symptom onset (57). According to our hypothesis, the immunosuppressive activities of IL-10 initially protect the lung from immune-mediated complications and, thereby, delay symptom onset, i.e., prolong the incubation period of SARS-CoV-2 infection. Concurrently, IL-10 impedes development of an efficient adaptive antiviral immune response, conniving at continued virus replication and thus an early viral load peak and prolonged shedding compared to SARS-CoV. As increased IL-10 was documented as early as in the first week after symptom onset (9), it would be attractive to also consider the mean duration from symptom onset to hospitalization as a proxy for further suppression of SARS-CoV-2 infection inflicted lung damage, potentially, through IL-10 that may delay the need for medical attention. For SARS patients, this period varied between 3 and 5 days (117), but for COVID-19 the reported numbers span a very wide range of 2.6–9.7 days (118) which so far precludes drawing conclusions.

One notable variation in COVID-19 pathology is that death from respiratory failure is not necessarily associated with severe lung damage but with a high load of viral RNA. In most recorded fatal cases, the immune response appears to yield to a cascade of detrimental inflammatory lymphoid and myeloid cell infiltration, complement activation, coagulopathy, and diffuse alveolar damage. In the two-phase model by Nienhold et al., however, the high-viral-load group and the lung-damage group represent death in the early and late phase of critical illness, respectively (26). In keeping with this model, the association of the increase in IL-10 with disease severity may indicate that the anti-inflammatory properties of IL-10 contribute to lung protection and virus replication not only in the pre-clinical phase, but that this cytokine may still be double-dealing in the critical phase of COVID-19 although its blood levels are already slowly declining.

In the following, we refer to the particular dynamics of IL-10 in peripheral blood during SARS-CoV-2 infection as an endotype (119) that we propose to be an etiological factor in lung protection and the limited efficiency to clear the virus. Accordingly, the lack of increased IL-10 in SARS was suggested before to favor immune-mediated lung damage (75) and represents a distinct endotype of a coronaviral infection. In short, we suspect two different endotypes to account for different transmission characteristics and clinical presentations, referred to here as clinical phenotypes, of the two viral infections.

### Possible Cellular Sources of IL-10 in COVID-19

Generally, different populations of innate immune cells can release IL-10 in early infection including pneumonia, whereas T cells, enclosing Tregs, and NK cells seem to dominate at later stages (107). Each of these populations was found to be functionally exhausted and/or reduced in peripheral blood of patients with severe COVID-19, which also applies to T cells in the lung. So far, an increase in IL-10 production has exclusively been associated with an increase in disease severity for a peripheral Treg population (115). In the SARS-CoV-2 infected respiratory tract, infiltrating myeloid cells and lymphoid cells enriched in the lower and NK cells and cytotoxic lymphocytes in the upper airways, in principle, represent candidate producers of IL-10 in the absence, however, of any data on IMs yet. Unfortunately, protein level evidence for intracellular production of cytokines in specific immune cell populations and for their secretion in the airways and other tissues is lacking. Therefore, we advocate the inclusion of IL-10 into future cytokine profiling efforts in cells and tissues of COVID-19 patients including the respiratory tract and the CNS.

### IL-10 as a Therapeutic Target in COVID-19?

IL-10 is predominantly thought of as a negative regulator of the initiation of an adaptive T cell response (78, 79). It is reasonable to assume that this acitivity counteracts hyperinflammation but also inhibits antiviral defense in COVID-19. Besides acting on antigen-presenting cells and T cells, IL-10 has also been demonstrated to stimulate expansion and cytokine production in murine mast cells (120). Some authors have theorized that mast cell activation syndrome contributes to hyperinflammation in severe COVID-19 and could also be a target for therapeutic inhibition (121). Yet, in the absence of direct evidence for this pro-inflammatory process in COVID-19, we here consider the established anti-inflammatory effects of IL-10 as critical for its proposed role in both lung protection and interference with viral clearance.

The net effect of therapeutic IL-10 signaling blockade in COVID-19, whether increased vulnerability of the lung to inflammation or desirable antiviral immunity, is difficult to predict. Antibody-mediated IL-6 receptor blockade, for instance, initially appeared to dampen hyperinflammation in patients with severe COVID-19 (122). But neither tocilizumab nor salirumab, two anti-IL-6 receptor monoclonal antibodies used to treat rheumatoid arthritis, has subsequently met expectations in randomized, double-blind, placebo-controlled phase 3 clinical trials (123, 124). They did not improve outcomes of critically ill patients which may caution against high expectations of targeting cytokine signaling in severe and critical COVID-19 more generally (125).

### Viral Genotype-Endotype-Clinical Phenotype Relationships

**Figure 1:**
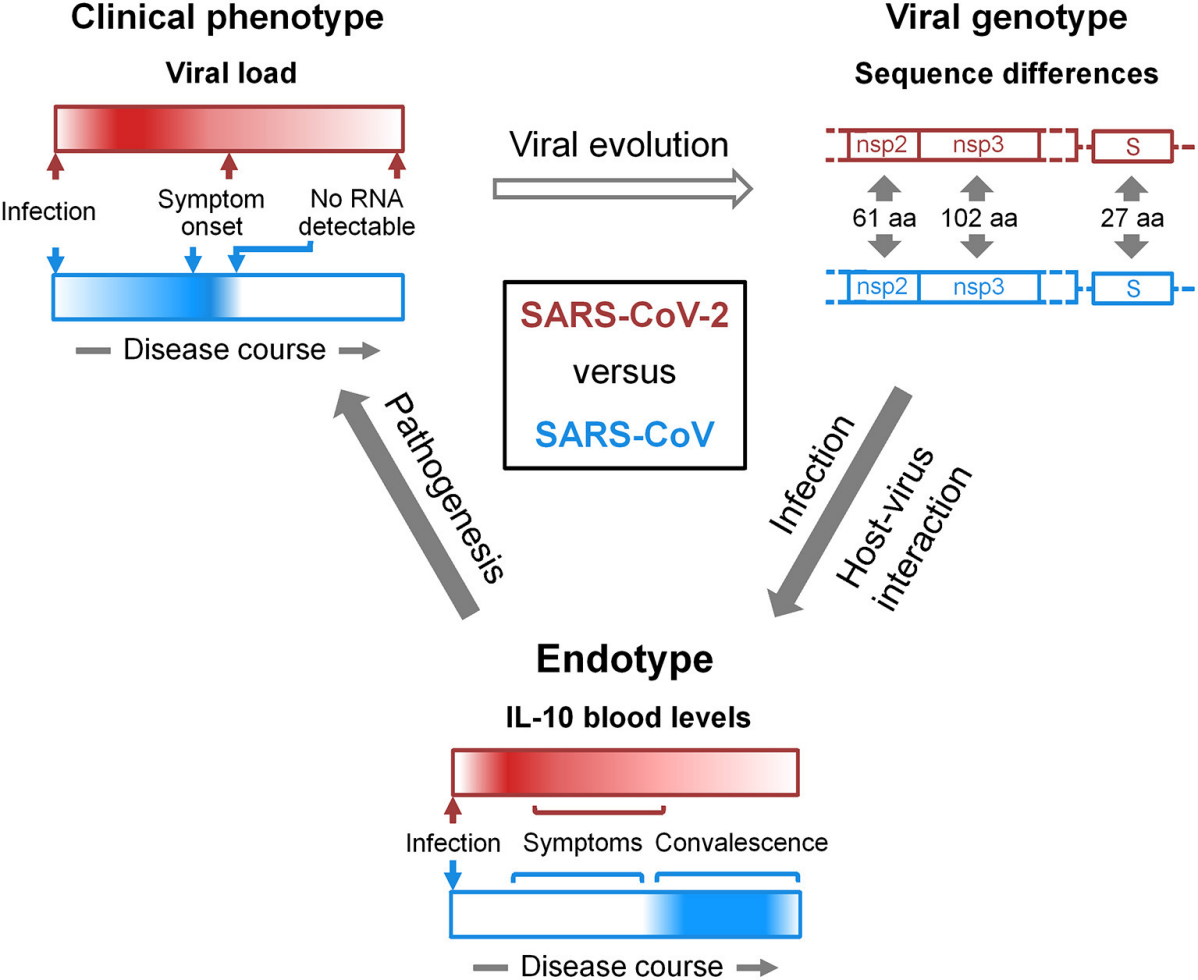
*Viral genotype-endotype-clinical phenotype* relationships in COVID-19 and SARS. At the **bottom**, the dynamics of the IL-10 response to SARS-CoV-2 (red) and SARS-CoV (blue) infections is schematized as horizontal bars. Dark color and white represent high and low levels, respectively, of IL-10 in peripheral blood (section IL-10 in COVID-19). In severe COVID-19 but not in SARS, IL-10 was found increased already in the first week of symptoms (9). Data on the levels of blood IL-10 in relation to the resolution of symptoms and in COVID-19-convalsecent blood has not yet been reported explicitly, but they appear to slowly decline with symptoms over the course of 2–3 weeks. We refer to IL-10 dynamics as *endotype*. In light of the lung protective properties of IL-10 and its role in microbial persistence (section Ambiguous Role of IL-10 in Lung Injury and Infection), we propose that the difference in this *endotype* contributes to the different viral load kinetics in COVID-19 and SARS (section SARS-CoV-2 Transmission vs. Clearance). Viral load kinetics are schematized at the **top left** in an analogous manner to the *endotype* and are referred to as *clinical phenotype*. The open arrow connecting *clinical phenotype* and *viral genotype* considers viral evolution in general inclusive of species jumping. The *genotype* schematic at the **top right** focuses on specific genome regions of the two viruses with sequence differences that give rise to the indicated numbers of amino acid (aa) changes in non-structural proteins 2 and 3 (nsp2, nsp3) and the spike (S) protein (section Discussion). One or a combination of such variations is likely responsible for the differential IL-10 response. However, a functional role of silent mutations cannot be excluded. It also has to be noted, that the annotation of open reading frames in the SARS-CoV-2 genome appears not yet complete, and new polypeptide products are being discovered (126). This type of diagram can be adapted in a flexible manner. For instance, it can be used to represent a variety of differences in *viral genotypes*, i.e., phylogenetic diversity, and putatively related *endo-* and *phenotypes* including those that may emerge during SARS-CoV-2 pandemic (127–130). Instead of the *viral genotype*, host genetic risk factors of COVID-19 (131–134) and their *endotype* and *phenotype* relations can be illustrated. Accordingly, *endotypes* can consist, for instance, in various immune evasion strategies and *clinical phenotypes* in any patient outcome. Moreover, external factors that influence evolution (top arrow), infection (right arrow), and pathogenesis (left arrow) can be incorporated, such as environmental influences.

New insight into the pathology of COVID-19 and support for antiviral strategies may be gained by comparing the clinical phenotypes and etiological endotypes of SARS-CoV-2 and SARS-CoV infections and identifying the underlying variation in the viral genotypes (Figure 1). Differences in clinical phenotypes consist in an earlier viral load peak and a longer duration of infectivity as well as overall lower severity in COVID-19 than in SARS. The corresponding differences in endotypes consist in an early increase in blood IL-10 in symptomatic COVID-19 patients compared to no increase in symptomatic SARS. Importantly, an early increase in IL-10 is neither a necessary precondition for progression to severe and critical illness nor is its absence for successful virus spread. Yet, we suspect the untimely IL-10 response to SARS-CoV-2 infection to promote immune evasion and virus replication, and to depend on an as yet unidentified viral factor. Conversely, it is possible that SARS-CoV, in contrast to SARS-CoV-2, actively suppresses the IL-10 response, interfering with resolution of inflammation and, thereby, aggravating disease earlier. Identification of the features of the viral genotypes and virus proximal host pathways underlying the differential IL-10 response to the two viruses may pinpoint therapeutic strategies that are more promising than the direct targeting of IL-10 signaling.

Coronaviruses use numerous strategies to evade the innate immune response (135). These include among others escaping RNA sensing, achieving host shut-off, and inhibiting stress granule formation and type 1 IFN signaling. The particular pandemic potential and virulence of SARS-CoV-2 has likely evolved through a combination of these and other endotypic traits. Coronaviruses are known to deploy and co-opt both their structural and non-structural proteins (nsps) and protein domains to evade the innate immune response. SARS-CoV-2 and SARS-CoV differ in about 21% of their genome sequences with, e.g., 61 amino acid substitutions in nsp2, 102 in nsp3, and 27 in the S protein (136). The search for the viral factors that account for the differences in clinical phenotypes and endotypes between SARS-CoV-2 and SARS-CoV infection should focus on these particular genomic regions. In practice, genotype-endotype-phenotype relationships (Figure 1) could be analyzed using recombinant viruses in animal models that replicate the differences in clinical phenotype as well as endotype, respectively, in this case the kinetics of viral load/lung damage and IL-10 dynamics during SARS-CoV-2 and SARS-CoV infections. Finally, understanding the genotype-endotype-phenotype relationships in coronaviral infections may aid predicting both outbreak risk and altered virulence from real time surveillance of coronavirus genetic diversity in natural reservoirs and in humans. Just as the human angiotensin-converting enzyme 2-compatible RBD, a major determinant of viral entry, was very likely already present for decades in bat coronaviruses (137) and is subject to continued mutation as seen in SARS-CoV-2 isolates obtained from humans (138), other as yet unrecognized genotypes that favor immune evasion and, thereby, disease in humans may already circulate.

## Limitations

As a limitation, our current hypothesis rests on clinical studies conducted mainly during the first wave of the SARS-CoV-2 pandemic, in the northern hemisphere, and with varying national containment strategies. In the absence of specific therapies, the off-label use of medications without proven benefit to outweigh risks, such as chloroquine and hydroxychloroquine (139), potentially, confounded results of some studies on COVID-19 patients. Also, an increase in the use of remdesivir and anticoagulants in high-risk patients and steroid use in critically ill patients may reduce comparability of studies from the beginning of the pandemic to more recent work.

## Conclusion

More population-based epidemiological and clinical surveillance is needed to substantiate our knowledge on the clinical phenotype of coronaviral diseases in general. The host response appears to be a major determinant of poor outcome in COVID-19, but much remains to learnt about the immediate host-virus interaction. Further understanding of the disease process (endotype) may be gained by comparative analysis of genotype-endotype-phenotype relationships (Figure 1). Here, we suspect that the increase in IL-10 constitutes a particular COVID-19 disease endotype that contributes to lung protection but also interferes with viral clearance very early following infection up to critical illness. We expect focused clinical analysis, above all the identification of the location and cellular source of IL-10 production, combined with the use of recombinant viruses in animal models that replicate the clinical phenotype of COVID-19 to reveal the underlying viral trigger and IL-10 dependent pathways. In these studies, the focus should not be limited to acute infection but furthermore consider convalescence, the emerging sequelae of COVID-19 (140), and the impact of vaccination. In conclusion, identifying the viral trigger of IL-10 release (viral genotype) and the cellular source and downstream pathways of IL-10 (endotype) in SARS-CoV-2 infection will reveal whether increased blood levels of this cytokine are a bystander or driver of disease in the different phases of COVID-19 (clinical phenotype). Last but not least, it is possible that IL-10 cooperates toward immunosuppression with other anti-inflammatory cytokines found increased in symptomatic COVID-19, such as IL-4, and IL-1RA (4, 6–9).

## Author Contributions

HL: conceptualization and writing (original manuscript). SV and MT: review and editing. TK: conceptualization and review. All authors contributed to the article and approved the submitted version.

## Conflict of Interest

The authors declare that the research was conducted in the absence of any commercial or financial relationships that could be construed as a potential conflict of interest.

## References

[1] Guan WJ Ni ZY Hu Y Liang WH Ou CQ He JX. Clinical characteristics of coronavirus disease 2019 in China. N Engl J Med. (2020) 382:1708–20. 10.1056/NEJMoa2002032 32109013 PMC7092819

[2] European Centre for Disease Prevention and Control. COVID-19 Situation Update Worldwide. (2020). Available online at: https://www.ecdc.europa.eu/en/geographical-distribution-2019-ncov-cases (accessed February 17, 2021).

[3] Maggi E Canonica GW Moretta L. COVID-19: unanswered questions on immune response and pathogenesis. J Allergy Clin Immunol. (2020) 146:18–22. 10.1016/j.jaci.2020.05.001 32389590 PMC7205667

[4] Huang C Wang Y Li X Ren L Zhao J Hu Y. Clinical features of patients infected with 2019 novel coronavirus in Wuhan, China. Lancet. (2020) 395:497–506. 10.1016/S0140-6736(20)30183-5 31986264 PMC7159299

[5] Yang Y Shen C Li J Yuan J Wei J Huang F. Plasma IP-10 and MCP-3 levels are highly associated with disease severity and predict the progression of COVID-19. J Allergy Clin Immunol. (2020) 146:119–27.e4. 10.1016/j.jaci.2020.04.027 32360286 PMC7189843

[6] Liu Y Zhang C Huang F Yang Y Wang F Yuan J. Elevated plasma levels of selective cytokines in COVID-19 patients reflect viral load and lung injury. Natl Sci Rev. (2020) 7:1003–11. 10.1093/nsr/nwaa037 PMC7107806 34676126

[7] Liu J Li S Liu J Liang B Wang X Wang H. Longitudinal characteristics of lymphocyte responses and cytokine profiles in the peripheral blood of SARS-CoV-2 infected patients. EBioMedicine. (2020) 55:102763. 10.1016/j.ebiom.2020.102763 32361250 PMC7165294

[8] Han H Ma Q Li C Liu R Zhao L Wang W. Profiling serum cytokines in COVID-19 patients reveals IL-6 and IL-10 are disease severity predictors. Emerg Microbes Infect. (2020) 9:1123–30. 10.1080/22221751.2020.1770129 32475230 PMC7473317

[9] Zhao Y Qin L Zhang P Li K Liang L Sun J. Longitudinal COVID-19 profiling associates IL-1RA and IL-10 with disease severity and RANTES with mild disease. JCI Insight. (2020) 5:e139834. 10.1172/jci.insight.139834 32501293 PMC7406242

[10] Hadjadj J Yatim N Barnabei L Corneau A Boussier J Smith N. Impaired type I interferon activity and exacerbated inflammatory responses in severe Covid-19 patients. Science. (2020) 369:718–24. 10.1126/science.abc6027 32661059 PMC7402632

[11] Qin C Zhou L Hu Z Zhang S Yang S Tao Y. Dysregulation of immune response in patients with coronavirus 2019 (COVID-19) in Wuhan, China. Clin Infect Dis. (2020) 71:762–68. 10.1093/cid/ciaa248 32161940 PMC7108125

[12] Sinha P Matthay MA Calfee CS. Is a “cytokine storm” relevant to COVID-19? JAMA Intern Med. (2020) 180:1152–54. 10.1001/jamainternmed.2020.3313 32602883

[13] Kox M Waalders NJB Kooistra EJ Gerretsen J Pickkers P. Cytokine levels in critically ill patients with COVID-19 and other conditions. JAMA. (2020) 324:1565–67 10.1001/jama.2020.17052 32880615 PMC7489366

[14] Jamilloux Y Henry T Belot A Viel S Fauter M El Jammal T. Should we stimulate or suppress immune responses in COVID-19? Cytokine and anti-cytokine interventions. Autoimmun Rev. (2020) 19:102567. 10.1016/j.autrev.2020.102567 32376392 PMC7196557

[15] McKechnie JL Blish CA. The innate immune system: fighting on the front lines or fanning the flames of COVID-19? Cell Host Microbe. (2020) 27:863–9. 10.1016/j.chom.2020.05.009 32464098 PMC7237895

[16] Costela-Ruiz VJ Illescas-Montes R Puerta-Puerta JM Ruiz C Melguizo-Rodriguez L. SARS-CoV-2 infection: the role of cytokines in COVID-19 disease. Cytokine Growth Factor Rev. (2020) 54:62–75. 10.1016/j.cytogfr.2020.06.001 32513566 PMC7265853

[17] Tay MZ Poh CM Renia L MacAry PA Ng LFP. The trinity of COVID-19: immunity, inflammation and intervention. Nat Rev Immunol. (2020) 20:363–74. 10.1038/s41577-020-0311-8 32346093 PMC7187672

[18] Diao B Wang C Tan Y Chen X Liu Y Ning L. Reduction and functional exhaustion of T cells in patients with coronavirus disease 2019 (COVID-19). Front Immunol. (2020) 11:827. 10.3389/fimmu.2020.00827 32425950 PMC7205903

[19] Zheng M Gao Y Wang G Song G Liu S Sun D. Functional exhaustion of antiviral lymphocytes in COVID-19 patients. Cell Mol Immunol. (2020) 17:533–5. 10.1038/s41423-020-0402-2 32203188 PMC7091858

[20] Mangalmurti N Hunter CA. Cytokine storms: understanding COVID-19. Immunity. (2020) 53:19–25. 10.1016/j.immuni.2020.06.017 32610079 PMC7321048

[21] Totura AL Baric RS. SARS coronavirus pathogenesis: host innate immune responses and viral antagonism of interferon. Curr Opin Virol. (2012) 2:264–75. 10.1016/j.coviro.2012.04.004 22572391 PMC7102726

[22] Schulte-Schrepping J Reusch N Paclik D Bassler K Schlickeiser S Zhang B. Severe COVID-19 is marked by a dysregulated myeloid cell compartment. Cell. (2020) 182:1419–40.e23. 10.1016/j.cell.2020.08.001 32810438 PMC7405822

[23] Broggi A Ghosh S Sposito B Spreafico R Balzarini F Lo Cascio A. Type III interferons disrupt the lung epithelial barrier upon viral recognition. Science. (2020) 369:706–12. 10.1126/science.abc3545 32527925 PMC7292499

[24] Zhou Z Ren L Zhang L Zhong J Xiao Y Jia Z. Heightened innate immune responses in the respiratory tract of COVID-19 patients. Cell Host Microbe. (2020) 27:883–90.e2. 10.1016/j.chom.2020.04.017 32407669 PMC7196896

[25] Bost P Giladi A Liu Y Bendjelal Y Xu G David E. Host-viral infection maps reveal signatures of severe COVID-19 patients. Cell. (2020) 181:1475–88.e12. 10.1016/j.cell.2020.05.006 32479746 PMC7205692

[26] Nienhold R Ciani Y Koelzer VH Tzankov A Haslbauer JD Menter T. Two distinct immunopathological profiles in autopsy lungs of COVID-19. Nat Commun. (2020) 11:5086. 10.1038/s41467-020-18854-2 33033248 PMC7546638

[27] Thiel V Weber F. Interferon and cytokine responses to SARS-coronavirus infection. Cytokine Growth Factor Rev. (2008) 19:121–32. 10.1016/j.cytogfr.2008.01.001 18321765 PMC7108449

[28] Zhou F Yu T Du R Fan G Liu Y Liu Z. Clinical course and risk factors for mortality of adult inpatients with COVID-19 in Wuhan, China: a retrospective cohort study. Lancet. (2020) 395:1054–62. 10.1016/S0140-6736(20)30566-3 32171076 PMC7270627

[29] Peruzzi B Bencini S Capone M Mazzoni A Maggi L Salvati L. Quantitative and qualitative alterations of circulating myeloid cells and plasmacytoid DC in SARS-CoV-2 infection. Immunology. (2020) 161:345–53. 10.1111/imm.13254 32870529 PMC7692244

[30] Remy KE Mazer M Striker DA Ellebedy AH Walton AH Unsinger J. Severe immunosuppression and not a cytokine storm characterizes COVID-19 infections. JCI Insight. (2020) 5:e140329. 10.1172/jci.insight.140329 32687484 PMC7526441

[31] Azkur AK Akdis M Azkur D Sokolowska M van de Veen W Bruggen MC. Immune response to SARS-CoV-2 and mechanisms of immunopathological changes in COVID-19. Allergy. (2020) 75:1564–81. 10.1111/all.14364 32396996 PMC7272948

[32] Singh Y Trautwein C Fendel R Krickeberg N Held J Kreidenweiss A. SARS-CoV-2 infection paralyzes cytotoxic and metabolic functions of immune cells. bioRxiv [Preprint]. (2020). 10.1101/2020.09.04.282780 PMC8159709 34075347

[33] Chua RL Lukassen S Trump S Hennig BP Wendisch D Pott F. COVID-19 severity correlates with airway epithelium-immune cell interactions identified by single-cell analysis. Nat Biotechnol. (2020) 38:970–9. 10.1038/s41587-020-0602-4 32591762

[34] Liao M Liu Y Yuan J Wen Y Xu G Zhao J. Single-cell landscape of bronchoalveolar immune cells in patients with COVID-19. Nat Med. (2020) 26:842–4. 10.1038/s41591-020-0901-9 32398875

[35] Kaneko N Kuo HH Boucau J Farmer JR Allard-Chamard H Mahajan VS. Loss of Bcl-6-expressing T follicular helper cells and germinal centers in COVID-19. Cell. (2020) 183:143–57.e13. 10.1016/j.cell.2020.08.025 32877699 PMC7437499

[36] Gu J Gong E Zhang B Zheng J Gao Z Zhong Y. Multiple organ infection and the pathogenesis of SARS. J Exp Med. (2005) 202:415–24. 10.1084/jem.20050828 16043521 PMC2213088

[37] Cameron MJ Bermejo-Martin JF Danesh A Muller MP Kelvin DJ. Human immunopathogenesis of severe acute respiratory syndrome (SARS). Virus Res. (2008) 133:13–9. 10.1016/j.virusres.2007.02.014 17374415 PMC7114310

[38] Su Y Chen D Yuan D Lausted C Choi J Dai CL. Multi-omics resolves a sharp disease-state shift between mild and moderate COVID-19. Cell. (2020) 183:1479–95. 10.1016/j.cell.2020.10.037 33171100 PMC7598382

[39] WHO Rapid Evidence Appraisal for COVID-19 Therapies (REACT) Working Group Sterne JAC Murthy S Diaz JV Slutsky AS Villar J. Association between administration of systemic corticosteroids and mortality among critically Ill patients with COVID-19: a meta-analysis. JAMA. (2020) 324:1330–41. 10.1001/jama.2020.17023 32876694 PMC7489434

[40] Polack FP Thomas SJ Kitchin N Absalon J Gurtman A Lockhart S. Safety and efficacy of the BNT162b2 mRNA Covid-19 vaccine. N Engl J Med. (2020) 383:2603–15. 10.1056/NEJMoa2034577 33301246 PMC7745181

[41] Baden LR El Sahly HM Essink B Kotloff K Frey S Novak R. Efficacy and safety of the mRNA-1273 SARS-CoV-2 vaccine. N Engl J Med. (2020) 384:403–16. 10.1056/NEJMoa2035389 33378609 PMC7787219

[42] World Health Organization. Draft Landscape of COVID-19 Candidate Vaccines. Available online at: https://www.who.int/publications/m/item/draft-landscape-of-covid-19-candidate-vaccines (accessed January 22, 2021).

[43] Fontanet A Cauchemez S. COVID-19 herd immunity: where are we? Nat Rev Immunol. (2020) 20:583–4. 10.1038/s41577-020-00451-5 32908300 PMC7480627

[44] Carmo A Pereira-Vaz J Mota V Mendes A Morais C da Silva AC. Clearance and persistence of SARS-CoV-2 RNA in patients with COVID-19. J Med Virol. (2020) 92:2227–31. 10.1002/jmv.26103 32484958 PMC7301002

[45] Lee S Kim T Lee E Lee C Kim H Rhee H. Clinical course and molecular viral shedding among asymptomatic and symptomatic patients with SARS-CoV-2 infection in a community treatment center in the Republic of Korea. JAMA Intern Med. (2020) 180:1–6. 10.1001/jamainternmed.2020.3862 32780793 PMC7411944

[46] Rhee C Kanjilal S Baker M Klompas M. Duration of severe acute respiratory syndrome coronavirus 2 (SARS-CoV-2) infectivity: when is it safe to discontinue isolation? Clin Infect Dis. (2020). 10.1093/cid/ciaa1249. [Epub ahead of print]. 33029620 PMC7499497

[47] Oran DP Topol EJ. Prevalence of asymptomatic SARS-CoV-2 infection : a narrative review. Ann Intern Med. (2020) 173:362–7. 10.7326/M20-3012 32491919 PMC7281624

[48] White EM Santostefano CM Feifer RA Kosar CM Blackman C Gravenstein S. Asymptomatic and presymptomatic severe acute respiratory syndrome coronavirus 2 infection rates in a multistate sample of skilled nursing facilities. JAMA Intern Med. (2020) 180:1709–11. 10.1001/jamainternmed.2020.5664 33074318 PMC7573793

[49] He X Lau EHY Wu P Deng X Wang J Hao X. Temporal dynamics in viral shedding and transmissibility of COVID-19. Nat Med. (2020) 26:672–5. 10.1038/s41591-020-0869-5 32296168

[50] Liu Y Centre for Mathematical Modelling of Infectious Diseases nCo VWG Funk S Flasche S. The contribution of pre-symptomatic infection to the transmission dynamics of COVID-2019. Wellcome Open Res. (2020) 5:58. 10.12688/wellcomeopenres.15788.1 32685697 PMC7324944

[51] Li R Pei S Chen B Song Y Zhang T Yang W. Substantial undocumented infection facilitates the rapid dissemination of novel coronavirus (SARS-CoV-2). Science. (2020) 368:489–93. 10.1126/science.abb3221 32179701 PMC7164387

[52] Johansson MA Quandelacy TM Kada S Prasad PV Steele M Brooks JT. SARS-CoV-2 transmission from people without COVID-19 symptoms. JAMA Netw Open. (2021) 4:e2035057. 10.1001/jamanetworkopen.2020.35057 33410879 PMC7791354

[53] Meyerowitz E Richterman A Bogoch I Low N Cevik M. Towards an accurate and systematic characterization of persistently asymptomatic infection with SARS-CoV-2. Lancet Infect Dis. (2020). 10.1016/S1473-3099(20)30837-9. [Epub ahead of print]. PMC7834404 33301725

[54] Moghadas SM Fitzpatrick MC Sah P Pandey A Shoukat A Singer BH. The implications of silent transmission for the control of COVID-19 outbreaks. Proc Natl Acad Sci USA. (2020) 117:17513–5. 10.1073/pnas.2008373117 32632012 PMC7395516

[55] Zaki N Mohamed EA. The estimations of the COVID-19 incubation period: a systematic review of the literature. medRxiv. (2020). 10.1101/2020.05.20.20108340 PMC7869687 33848893

[56] Lessler J Reich NG Brookmeyer R Perl TM Nelson KE Cummings DA. Incubation periods of acute respiratory viral infections: a systematic review. Lancet Infect Dis. (2009) 9:291–300. 10.1016/S1473-3099(09)70069-6 19393959 PMC4327893

[57] Benefield AE Skrip LA Clement A Althouse RA Chang S Althouse BM. SARS-CoV-2 viral load peaks prior to symptom onset: a systematic review and individual-pooled analysis of coronavirus viral load from 66 studies. medRxiv [Preprint]. (2020). 10.1101/2020.09.28.20202028

[58] Peiris JS Chu CM Cheng VC Chan KS Hung IF Poon LL. Clinical progression and viral load in a community outbreak of coronavirus-associated SARS pneumonia: a prospective study. Lancet. (2003) 361:1767–72. 10.1016/S0140-6736(03)13412-5 12781535 PMC7112410

[59] Cevik M Tate M Lloyd O Maraolo AE Schafers J Ho A. SARS-CoV-2, SARS-CoV-1 and MERS-CoV viral load dynamics, duration of viral shedding and infectiousness: a living systematic review and meta-analysis. Lancet Microbe. (2021) 2:e13–22. 10.2139/ssrn.3677918 33521734 PMC7837230

[60] Walsh KA Spillane S Comber L Cardwell K Harrington P Connell J. The duration of infectiousness of individuals infected with SARS-CoV-2. J Infect. (2020) 81:847–56. 10.1016/j.jinf.2020.10.009 33049331 PMC7547320

[61] Sessa F Bertozzi G Cipolloni L Baldari B Cantatore S D'Errico S. Clinical-forensic autopsy findings to defeat COVID-19 disease: a literature review. J Clin Med. (2020) 9:2026. 10.3390/jcm9072026 32605192 PMC7409028

[62] Davies NG Klepac P Liu Y Prem K Jit M group CC-w. Age-dependent effects in the transmission and control of COVID-19 epidemics. Nat Med. (2020) 26:1205–11. 10.1038/s41591-020-0962-9 32546824

[63] Ludvigsson JF. Systematic review of COVID-19 in children shows milder cases and a better prognosis than adults. Acta Paediatr. (2020) 109:1088–95. 10.1111/apa.15270 32202343 PMC7228328

[64] Kam KQ Yung CF Cui L Lin Tzer Pin R Mak TM Maiwald M. A well infant with coronavirus disease 2019 (COVID-19) with high viral load. Clin Infect Dis. (2020) 71:847–9. 10.1093/cid/ciaa201 32112082 PMC7358675

[65] Jones TC Mühlemann B Veith T Biele G Zuchowski M Hoffmann J. An analysis of SARS-CoV-2 viral load by patient age. medRxiv [Preprint]. (2020). 10.1101/2020.06.08.20125484

[66] Dong Y Mo X Hu Y Qi X Jiang F Jiang Z. Epidemiology of COVID-19 among children in China. Pediatrics. (2020) 145:e20200702. 10.1542/peds.2020-0702 32179660

[67] Gotzinger F Santiago-Garcia B Noguera-Julian A Lanaspa M Lancella L Calo Carducci FI. COVID-19 in children and adolescents in Europe: a multinational, multicentre cohort study. Lancet Child Adolesc Health. (2020) 4:653–61. 10.1016/S2352-4642(20)30177-2 32593339 PMC7316447

[68] Sun D Chen X Li H Lu XX Xiao H Zhang FR. SARS-CoV-2 infection in infants under 1 year of age in Wuhan City, China. World J Pediatr. (2020) 16:260–6. 10.1007/s12519-020-00368-y 32504360 PMC7274073

[69] Wu H Zhu H Yuan C Yao C Luo W Shen X. Clinical and immune features of hospitalized pediatric patients with coronavirus disease 2019 (COVID-19) in Wuhan, China. JAMA Netw Open. (2020) 3:e2010895. 10.1001/jamanetworkopen.2020.10895 32492165 PMC7272117

[70] van Doremalen N Lambe T Spencer A Belij-Rammerstorfer S Purushotham JN Port JR. ChAdOx1 nCoV-19 vaccination prevents SARS-CoV-2 pneumonia in rhesus macaques. Nature. (2020) 586:578–82. 10.1038/s41586-020-2608-y 32731258 PMC8436420

[71] McElvaney OJ McEvoy NL McElvaney OF Carroll TP Murphy MP Dunlea DM. Characterization of the inflammatory response to severe COVID-19 illness. Am J Respir Crit Care Med. (2020) 202:812–21. 10.1164/rccm.202005-1583OC 32584597 PMC7491404

[72] Lucas C Wong P Klein J Castro TBR Silva J Sundaram M. Longitudinal analyses reveal immunological misfiring in severe COVID-19. Nature. (2020) 584:463–69. 10.1038/s41586-020-2588-y 32717743 PMC7477538

[73] Hue S Beldi-Ferchiou A Bendib I Surenaud M Fourati S Frapard T. Uncontrolled innate and impaired adaptive immune responses in patients with COVID-19 acute respiratory distress syndrome. Am J Respir Crit Care Med. (2020) 202:1509–19. 10.1164/rccm.202005-1885OC 32866033 PMC7706149

[74] Wong CK Lam CW Wu AK Ip WK Lee NL Chan IH. Plasma inflammatory cytokines and chemokines in severe acute respiratory syndrome. Clin Exp Immunol. (2004) 136:95–103. 10.1111/j.1365-2249.2004.02415.x 15030519 PMC1808997

[75] Chien JY Hsueh PR Cheng WC Yu CJ Yang PC. Temporal changes in cytokine/chemokine profiles and pulmonary involvement in severe acute respiratory syndrome. Respirology. (2006) 11:715–22. 10.1111/j.1440-1843.2006.00942.x 17052299 PMC7192207

[76] Huang KJ Su IJ Theron M Wu YC Lai SK Liu CC. An interferon-gamma-related cytokine storm in SARS patients. J Med Virol. (2005) 75:185–94. 10.1002/jmv.20255 15602737 PMC7166886

[77] Zhang Y Li J Zhan Y Wu L Yu X Zhang W. Analysis of serum cytokines in patients with severe acute respiratory syndrome. Infect Immun. (2004) 72:4410–5. 10.1128/IAI.72.8.4410-4415.2004 15271897 PMC470699

[78] Saraiva M Vieira P O'Garra A. Biology and therapeutic potential of interleukin-10. J Exp Med. (2020) 217:e20190418. 10.1084/jem.20190418 31611251 PMC7037253

[79] Ouyang W O'Garra A. IL-10 family cytokines IL-10 and IL-22: from basic science to clinical translation. Immunity. (2019) 50:871–91. 10.1016/j.immuni.2019.03.020 30995504

[80] Dong Y Dai T Liu J Zhang L Zhou F. Coronavirus in continuous flux: from SARS-CoV to SARS-CoV-2. Adv Sci. (2020) 7:2001474. 10.1002/advs.202001474 32837848 PMC7361144

[81] Hofstetter C Flondor M Hoegl S Muhl H Zwissler B. Interleukin-10 aerosol reduces proinflammatory mediators in bronchoalveolar fluid of endotoxemic rat. Crit Care Med. (2005) 33:2317–22. 10.1097/01.CCM.0000182815.78568.B2 16215387

[82] Hoegl S Boost KA Czerwonka H Dolfen A Scheiermann P Muhl H. Inhaled IL-10 reduces biotrauma and mortality in a model of ventilator-induced lung injury. Respir Med. (2009) 103:463–70. 10.1016/j.rmed.2008.09.020 19006658

[83] Li HD Zhang QX Mao Z Xu XJ Li NY Zhang H. Exogenous interleukin-10 attenuates hyperoxia-induced acute lung injury in mice. Exp Physiol. (2015) 100:331–40. 10.1113/expphysiol.2014.083337 25480159

[84] Kawano H Kayama H Nakama T Hashimoto T Umemoto E Takeda K. IL-10-producing lung interstitial macrophages prevent neutrophilic asthma. Int Immunol. (2016) 28:489–501. 10.1093/intimm/dxw012 26976823

[85] Boehler A. The role of interleukin-10 in lung transplantation. Transpl Immunol. (2002) 9:121–4. 10.1016/S0966-3274(02)00045-X 12180818

[86] Chmiel JF Konstan MW Knesebeck JE Hilliard JB Bonfield TL Dawson DV. IL-10 attenuates excessive inflammation in chronic Pseudomonas infection in mice. Am J Respir Crit Care Med. (1999) 160:2040–7. 10.1164/ajrccm.160.6.9901043 10588626

[87] Sun J Madan R Karp CL Braciale TJ. Effector T cells control lung inflammation during acute influenza virus infection by producing IL-10. Nat Med. (2009) 15:277–84. 10.1038/nm.1929 19234462 PMC2693210

[88] McKinstry KK Strutt TM Buck A Curtis JD Dibble JP Huston G. IL-10 deficiency unleashes an influenza-specific Th17 response and enhances survival against high-dose challenge. J Immunol. (2009) 182:7353–63. 10.4049/jimmunol.0900657 19494257 PMC2724021

[89] van der Sluijs KF van Elden LJ Nijhuis M Schuurman R Pater JM Florquin S. IL-10 is an important mediator of the enhanced susceptibility to pneumococcal pneumonia after influenza infection. J Immunol. (2004) 172:7603–9. 10.4049/jimmunol.172.12.7603 15187140

[90] Sun K Torres L Metzger DW. A detrimental effect of interleukin-10 on protective pulmonary humoral immunity during primary influenza A virus infection. J Virol. (2010) 84:5007–14. 10.1128/JVI.02408-09 20200252 PMC2863832

[91] Bedoya F Cheng GS Leibow A Zakhary N Weissler K Garcia V. Viral antigen induces differentiation of Foxp3+ natural regulatory T cells in influenza virus-infected mice. J Immunol. (2013) 190:6115–25. 10.4049/jimmunol.1203302 23667113 PMC3703618

[92] Barthelemy A Ivanov S Fontaine J Soulard D Bouabe H Paget C. Influenza A virus-induced release of interleukin-10 inhibits the anti-microbial activities of invariant natural killer T cells during invasive pneumococcal superinfection. Mucosal Immunol. (2017) 10:460–9. 10.1038/mi.2016.49 27220813

[93] Mege JL Meghari S Honstettre A Capo C Raoult D. The two faces of interleukin 10 in human infectious diseases. Lancet Infect Dis. (2006) 6:557–69. 10.1016/S1473-3099(06)70577-1 16931407

[94] Sarikonda G von Herrath MG. Immunosuppressive mechanisms during viral infectious diseases. Methods Mol Biol. (2011) 677:431–47. 10.1007/978-1-60761-869-0_27 20941625 PMC7120576

[95] Wilson EB Brooks DG. The role of IL-10 in regulating immunity to persistent viral infections. Curr Top Microbiol Immunol. (2011) 350:39–65. 10.1007/82_2010_96 20703965 PMC3492216

[96] Kumar R Ng S Engwerda C. The role of IL-10 in malaria: a double edged sword. Front Immunol. (2019) 10:229. 10.3389/fimmu.2019.00229 30809232 PMC6379449

[97] Redford PS Murray PJ O'Garra A. The role of IL-10 in immune regulation *during M. tuberculosis* infection. Mucosal Immunol. (2011) 4:261–70. 10.1038/mi.2011.7 21451501

[98] Ring S Eggers L Behrends J Wutkowski A Schwudke D Kroger A. Blocking IL-10 receptor signaling ameliorates *Mycobacterium tuberculosis* infection during influenza-induced exacerbation. JCI Insight. (2019) 4:e126533. 10.1172/jci.insight.126533 30998505 PMC6542649

[99] Savarin C Bergmann CC. Fine tuning the cytokine storm by IFN and IL-10 following neurotropic coronavirus encephalomyelitis. Front Immunol. (2018) 9:3022. 10.3389/fimmu.2018.03022 30619363 PMC6306494

[100] Hu J Jolkkonen J Zhao C. Neurotropism of SARS-CoV-2 and its neuropathological alterations: similarities with other coronaviruses. Neurosci Biobehav Rev. (2020) 119:184–93. 10.1016/j.neubiorev.2020.10.012 33091416 PMC7571477

[101] Matschke J Lütgehetmann M Hagel C Sperhake JP Schröder AS Edle C. Neuropathology of patients with COVID-19 in Germany: a post-mortem case series. Lancet Neurol. (2020) 19:919–29. 10.1016/S1474-4422(20)30308-2 33031735 PMC7535629

[102] Mazer M Unsinger J Drewry A Walton A Osborne D Blood T. IL-10 has differential effects on the innate and adaptive immune systems of septic patients. J Immunol. (2019) 203:2088–99. 10.4049/jimmunol.1900637 31501258 PMC7206829

[103] Rubtsov YP Rasmussen JP Chi EY Fontenot J Castelli L Ye X. Regulatory T cell-derived interleukin-10 limits inflammation at environmental interfaces. Immunity. (2008) 28:546–58. 10.1016/j.immuni.2008.02.017 18387831

[104] Cope A Le Friec G Cardone J Kemper C. The Th1 life cycle: molecular control of IFN-gamma to IL-10 switching. Trends Immunol. (2011) 32:278–86. 10.1016/j.it.2011.03.010 21531623

[105] Perlman S Zhao J. Roles of regulatory T cells and IL-10 in virus-induced demyelination. J Neuroimmunol. (2017) 308:6–11. 10.1016/j.jneuroim.2017.01.001 28065579 PMC5474348

[106] Ciurkiewicz M Herder V Beineke A. Beneficial and detrimental effects of regulatory T cells in neurotropic virus infections. Int J Mol Sci. (2020) 21:1705. 10.3390/ijms21051705 PMC7084400 32131483

[107] Rojas JM Avia M Martin V Sevilla N. IL-10: a multifunctional cytokine in viral infections. J Immunol Res. (2017) 2017:6104054. 10.1155/2017/6104054 28316998 PMC5337865

[108] Nakajima H Gleich GJ Kita H. Constitutive production of IL-4 and IL-10 and stimulated production of IL-8 by normal peripheral blood eosinophils. J Immunol. (1996) 156:4859–66. 8648135

[109] Bedoret D Wallemacq H Marichal T Desmet C Quesada Calvo F Henry E. Lung interstitial macrophages alter dendritic cell functions to prevent airway allergy in mice. J Clin Invest. (2009) 119:3723–38. 10.1172/JCI39717 19907079 PMC2786798

[110] Toussaint M Fievez L Drion PV Cataldo D Bureau F Lekeux P. Myeloid hypoxia-inducible factor 1alpha prevents airway allergy in mice through macrophage-mediated immunoregulation. Mucosal Immunol. (2013) 6:485–97. 10.1038/mi.2012.88 22968421

[111] Sabatel C Radermecker C Fievez L Paulissen G Chakarov S Fernandes C. Exposure to bacterial CpG DNA protects from airway allergic inflammation by expanding regulatory lung interstitial macrophages. Immunity. (2017) 46:457–73. 10.1016/j.immuni.2017.02.016 28329706

[112] Hoppstadter J Diesel B Zarbock R Breinig T Monz D Koch M. Differential cell reaction upon Toll-like receptor 4 and 9 activation in human alveolar and lung interstitial macrophages. Respir Res. (2010) 11:124. 10.1186/1465-9921-11-124 20843333 PMC2949727

[113] Ural BB Yeung ST Damani-Yokota P Devlin JC de Vries M Vera-Licona P. Identification of a nerve-associated, lung-resident interstitial macrophage subset with distinct localization and immunoregulatory properties. Sci Immunol. (2020) 5:eaax8756. 10.1126/sciimmunol.aax8756 32220976 PMC7717505

[114] Iwata Y Matsushita T Horikawa M Dilillo DJ Yanaba K Venturi GM. Characterization of a rare IL-10-competent B-cell subset in humans that parallels mouse regulatory B10 cells. Blood. (2011) 117:530–41. 10.1182/blood-2010-07-294249 20962324 PMC3031478

[115] Neumann J Prezzemolo T Vanderbeke L Roca CP Gerbaux M Janssens S. An open resource for T cell phenotype changes in COVID-19 identifies IL-10-producing regulatory T cells as characteristic of severe cases. Clin Transl Immunol. (2020) 9:e1204. 10.1002/cti2.1204 PMC7662088 33209300

[116] Barton LM Duval EJ Stroberg E Ghosh S Mukhopadhyay S. COVID-19 autopsies, Oklahoma, USA. Am J Clin Pathol. (2020) 153:725–33. 10.1093/ajcp/aqaa062 32275742 PMC7184436

[117] Donnelly CA Ghani AC Leung GM Hedley AJ Fraser C Riley S. Epidemiological determinants of spread of causal agent of severe acute respiratory syndrome in Hong Kong. Lancet. (2003) 361:1761–6. 10.1016/S0140-6736(03)13410-1 12781533 PMC7112380

[118] Faes C Abrams S Van Beckhoven D Meyfroidt G Vlieghe E Hens N. Time between symptom onset, hospitalisation and recovery or death: statistical analysis of Belgian COVID-19 patients. Int J Environ Res Public Health. (2020) 17:7560. 10.3390/ijerph17207560 33080869 PMC7589278

[119] Anderson GP. Endotyping asthma: new insights into key pathogenic mechanisms in a complex, heterogeneous disease. Lancet. (2008) 372:1107–19. 10.1016/S0140-6736(08)61452-X 18805339

[120] Polukort SH Rovatti J Carlson L Thompson C Ser-Dolansky J Kinney SR. IL-10 enhances IgE-mediated mast cell responses and is essential for the development of experimental food allergy in IL-10-deficient mice. J Immunol. (2016) 196:4865–76. 10.4049/jimmunol.1600066 27183617 PMC4893936

[121] Afrin LB Weinstock LB Molderings GJ. Covid-19 hyperinflammation and post-Covid-19 illness may be rooted in mast cell activation syndrome. Int J Infect Dis. (2020) 100:327–32. 10.1016/j.ijid.2020.09.016 32920235 PMC7529115

[122] Xu X Han M Li T Sun W Wang D Fu B. Effective treatment of severe COVID-19 patients with tocilizumab. Proc Natl Acad Sci USA. (2020) 117:10970–5. 10.1073/pnas.2005615117 32350134 PMC7245089

[123] Parr JB. Time to reassess tocilizumab's role in COVID-19 pneumonia. JAMA Intern Med. (2021) 181:12–5. 10.1001/jamainternmed.2020.6557 33079980

[124] Sanofi. Available online at: https://www.sanofi.com/en/media-room/press-releases/2020/2020-07-02-22-30-00 (accessed July 2, 2020).

[125] Snow TAC Singer M Arulkumaran N. Immunomodulators in COVID-19: two sides to every coin. Am J Respir Crit Care Med. (2020) 202:1460–61. 10.1164/rccm.202008-3148LE 32926809 PMC7667893

[126] Finkel Y Mizrahi O Nachshon A Weingarten-Gabbay S Morgenstern D Yahalom-Ronen Y. The coding capacity of SARS-CoV-2. Nature. (2020) 589:125–30. 10.1038/s41586-020-2739-1 32906143

[127] Grubaugh ND Hanage WP Rasmussen AL. Making sense of mutation: what D614G means for the COVID-19 pandemic remains unclear. Cell. (2020) 182:794–5. 10.1016/j.cell.2020.06.040 32697970 PMC7332445

[128] Pachetti M Marini B Benedetti F Giudici F Mauro E Storici P. Emerging SARS-CoV-2 mutation hot spots include a novel RNA-dependent-RNA polymerase variant. J Transl Med. (2020) 18:179. 10.1186/s12967-020-02344-6 32321524 PMC7174922

[129] Pereira F. Evolutionary dynamics of the SARS-CoV-2 ORF8 accessory gene. Infect Genet Evol. (2020) 85:104525. 10.1016/j.meegid.2020.104525 32890763 PMC7467077

[130] Rambaut A Holmes EC O'Toole Á Hill V McCrone JT Ruis C. A dynamic nomenclature proposal for SARS-CoV-2 lineages to assist genomic epidemiology. Nat Microbiol. (2020) 5:1403–7. 10.1038/s41564-020-0770-5 32669681 PMC7610519

[131] Zeberg H Paabo S. The major genetic risk factor for severe COVID-19 is inherited from Neanderthals. Nature. (2020) 587:610–12. 10.1038/s41586-020-2818-3 32998156

[132] van der Made CI Simons A Schuurs-Hoeijmakers J van den Heuvel G Mantere T Kersten S. Presence of genetic variants among young men with severe COVID-19. JAMA. (2020) 324:663–73. 10.1001/jama.2020.13719 32706371 PMC7382021

[133] Beck DB Aksentijevich I. Susceptibility to severe COVID-19. Science. (2020) 370:404–5. 10.1126/science.abe7591 33093097

[134] Karaderi T Bareke H Kunter I Seytanoglu A Cagnan I Balci D. Host genetics at the intersection of autoimmunity and COVID-19: a potential key for heterogeneous COVID-19 severity. Front Immunol. (2020) 11:3314. 10.3389/fimmu.2020.586111 33414783 PMC7783411

[135] Kikkert M. Innate immune evasion by human respiratory RNA viruses. J Innate Immun. (2020) 12:4–20. 10.1159/000503030 31610541 PMC6959104

[136] Wu A Peng Y Huang B Ding X Wang X Niu P. Genome composition and divergence of the novel coronavirus (2019-nCoV) originating in China. Cell Host Microbe. (2020) 27:325–8. 10.1016/j.chom.2020.02.001 32035028 PMC7154514

[137] Boni MF Lemey P Jiang X Lam TT Perry BW Castoe TA. Evolutionary origins of the SARS-CoV-2 sarbecovirus lineage responsible for the COVID-19 pandemic. Nat Microbiol. (2020) 5:1408–17. 10.1038/s41564-020-0771-4 32724171

[138] Lauring AS Hodcroft EB. Genetic variants of SARS-CoV-2—What do they mean? JAMA. (2021) 325:529–31. 10.1001/jama.2020.27124 33404586

[139] U.S. Food and Drug Administration. Coronavirus (COVID-19) Update: FDA Revokes Emergency Use Authorization for Chloroquine and Hydroxychloroquine. (2020). Available online at: https://www.fda.gov/news-events/press-announcements/coronavirus-covid-19-update-fda-revokes-emergency-use-authorization-chloroquine-and (accessed February 17, 2021).

[140] Huang C Huang L Wang Y Li X Ren L Gu X. 6-month consequences of COVID-19 in patients discharged from hospital: a cohort study. Lancet. (2021) 397:220–32. 10.1016/S0140-6736(20)32656-8 33428867 PMC7833295

